# 

**DOI:** 10.1192/bjb.2024.98

**Published:** 2025-02

**Authors:** Rachel Bannister

**Affiliations:** Works as an Expert by Experience in several roles across the Royal College of Psychiatrists, London, UK and is co-founder and chair of the grassroots charity Mental Health – Time for Action Foundation. Email: mhtimeforaction@gmail.com

**Figure:**
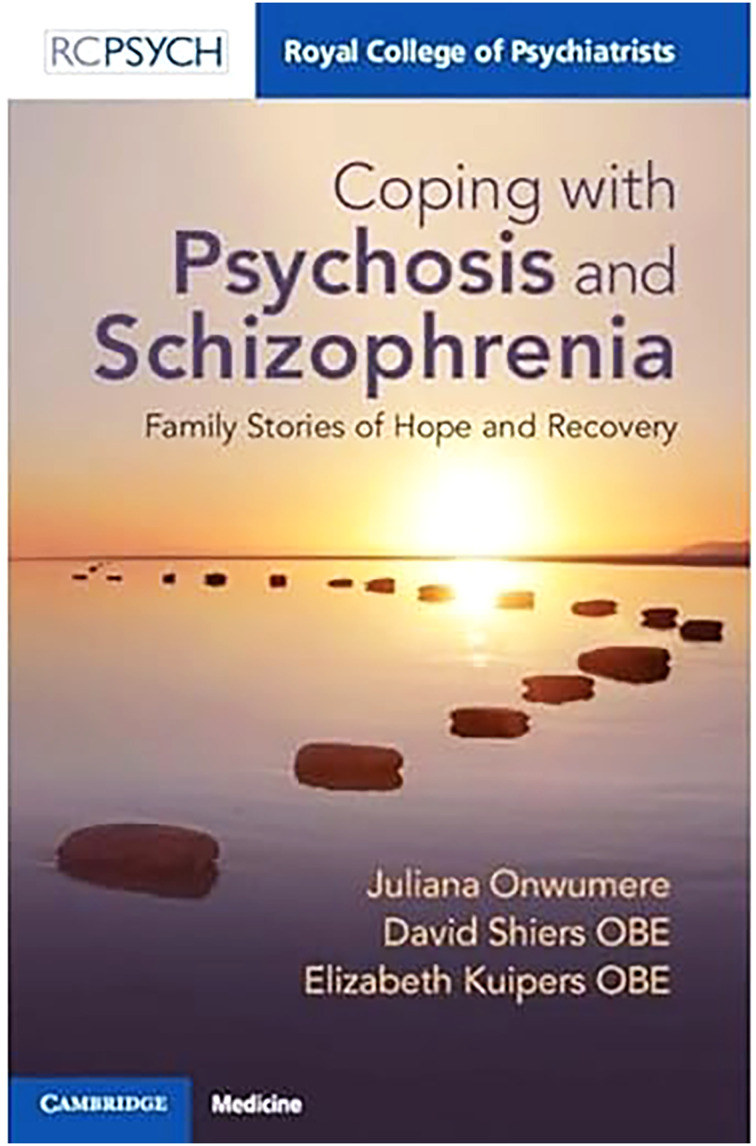

As an individual who has gained invaluable insights through direct experience in supporting a family member facing substantial mental health challenges for over a decade, I approached this work with a deep sense of understanding and empathy. The compilation of narratives reflecting diverse viewpoints – those of parents, siblings and partners – I found both compelling and emotionally impactful.

There is a notable sense of hope and positivity within these family testimonials regarding the care of relatives experiencing psychosis. A particularly salient point is the significant benefit derived from connecting with others in analogous circumstances. Chris and Terry aptly articulate it as follows: ‘Talking to other carers was the single most important thing we did’ (p. 25). Annette, a parental caregiver, remarked ‘Attending the carers’ group made me realise that there was support available for myself and my family’ (p. 14). From my own experiences, I can attest to the critical importance of establishing connections with others encountering similar situations. Annette recounts that it was only after engaging with fellow caregivers that she was able to relinquish the self-blame she felt regarding her son's situation. This struggle with self-blame is a common phenomenon, and it is often through mutual support that individuals can come to realise they are not at fault.

The accounts detailing the lack of inclusion of family members in treatment are unlikely to surprise those facing comparable predicaments. It is therefore disappointing to note the omission of any reference in the initial chapter to the social and family network approach known as Open Dialogue, which has recently been the focus of the largest randomised controlled trial in mental health within NHS England. Although the results of this trial are still pending publication, there is a considerable body of evidence globally highlighting the positive outcomes associated with this person-centred, holistic approach.

Under the ‘Causes' section in Chapter 1, ‘Understanding Psychosis and Schizophrenia’, it is regrettable that there is merely a cursory mention of trauma as a contributing factor in psychosis and schizophrenia. Although the authors present data indicating an elevated risk of psychosis among racial and ethnic minority and migrant communities, it would have been beneficial to delve deeper into the research evidence on underlying factors, including the influences of racialised trauma and socioeconomic challenges.

I am optimistic that these personal narratives will offer valuable insights and comfort to individuals accompanying a family member through the challenging journey of managing psychosis or schizophrenia. Nevertheless, I departed from the book with a sense of disappointment regarding the emphasis placed in the introductory chapter on medication as the principal form of treatment. This emphasis reinforces the notion that early intervention with medication during a psychotic episode is critical, with the implication that failure to do so could lead to exacerbation of their illness and negative repercussions. It is my understanding that this perspective is a topic of considerable debate within the field.

This volume succeeds in illuminating the complex and emotional journey of caregiving for loved ones experiencing mental health challenges. The narratives bring to life the importance of connection and shared experiences, something I can personally attest to through my own experiences. The book's message of hope and the vital role of support from others in similar situations are powerful takeaways. However, I felt it missed opportunities to address broader, evidence-based approaches. Despite these limitations, the collection of stories will undoubtedly offer comfort, guidance and a sense of solidarity to families navigating similar paths.

